# Respiratory Support Effects over Time on Regional Lung Ventilation Assessed by Electrical Impedance Tomography in Premature Infants

**DOI:** 10.3390/medicina60030494

**Published:** 2024-03-17

**Authors:** Ernestas Virsilas, Arunas Valiulis, Raimondas Kubilius, Skaiste Peciuliene, Arunas Liubsys

**Affiliations:** 1 Clinic of Children’s Diseases, Institute of Clinical Medicine, Vilnius University Faculty of Medicine, LT-03101 Vilnius, Lithuania; 2 Department of Rehabilitation, Lithuanian University of Health Sciences, LT-50161 Kaunas, Lithuania

**Keywords:** respiratory distress syndrome, mechanical ventilation, high-flow nasal cannula, continuous positive airway pressure, electrical impedance tomography, preterm newborns

## Abstract

*Background and objectives:* Respiratory distress syndrome (RDS) frequently necessitates respiratory support. While non-invasive methods are typically the preferred approach, mechanical ventilation becomes necessary for patients with insufficient response. Our study aimed to compare two common respiratory support modes, volume-targeted mechanical ventilation and non-invasive ventilation continuous positive airway pressure (CPAP) and high-flow nasal cannula (HFNC), using electrical impedance tomography. *Materials and Methods*: Infants with very low birth weight and gestational ages of less than 32 weeks were eligible for inclusion in the study. All enrolled infants were beyond the transitional period (>72 h of age). The infants were divided into two groups: infants receiving invasive respiratory support through an endotracheal tube and infants receiving non-invasive respiratory support. We used electrical impedance tomography to assess end-expiratory lung impedance (EELZ), DeltaZ, heterogeneity, and regional ventilation distribution. Patients were evaluated at 0, 30, and 60 min after assuming the supine position to examine potential time-related effects. *Results*: Our study initially enrolled 97 infants, and the final analysis included a cohort of 72 infants. Ventilated infants exhibited significantly larger EELZ compared to their non-invasive counterparts (*p* = 0.026). DeltaZ was also greater in the invasive respiratory support group (*p* < 0.001). Heterogeneity was higher in the non-invasive group and did not change significantly over time. The non-invasive group demonstrated significantly greater ventilation in the dependent lung areas compared to intubated patients (*p* = 0.005). Regional distribution in the left lung was lower than in the right lung in both groups; however, this difference was significantly more pronounced in intubated patients (*p* < 0.001). *Conclusions*: Our study revealed that volume-targeted mechanical ventilation results in higher EELZ and DeltaZ compared to spontaneously breathing infants receiving non-invasive respiratory support. However, lung heterogeneity was lower during mechanical ventilation. Our study also reaffirmed that spontaneous breathing promotes greater involvement of the dependent lung compared to mechanical ventilation.

## 1. Introduction

Respiratory distress syndrome (RDS) is frequently observed in preterm infants, particularly those born very preterm or extremely preterm. The predominant factor leading to RDS is a deficiency in surfactant, resulting from inadequate production by type II pneumocytes. Additionally, the condition is compounded by an excessively compliant chest wall. As a consequence of this respiratory insufficiency, the majority of infants in these gestational categories require various forms of respiratory support. While non-invasive respiratory support is recommended initially, mechanical ventilation is still frequently used in those who do not adequately respond or fail to respond to the former [1]. For such occasions, “gentle” ventilation strategies are recommended. ‘Gentle’ ventilation involves administering respiratory support in a manner aimed at minimizing potential harm or trauma to the lungs. Despite the widespread adoption of pressure-limited, volume-targeted ventilation practices in many healthcare centers [2], it is important to note that this approach is still associated with the risk of ventilator-induced lung injury (VILI) [3]. Theoretically, in order to avoid significant lung damage, clinicians must follow several fundamental principles of ventilation: optimize lung recruitment (open lung ventilation) by producing adequate functional residual capacity (FRC) (1), prevent volutrauma by avoiding excessive tidal volumes (TV) (2) and prevent or reverse atelectasis (3) by providing adequate positive end-expiratory pressure (PEEP) [4]. Yet, in practice, identifying the optimal parameters for individual patients proves to be a challenging endeavor, often necessitating substantial adjustments even after the recommended values have been implemented. This inherent challenge arises from the delicate balance required to achieve optimal ventilation. Adequate FRC, TV, and PEEP are essential for effective ventilation, yet deviations from the appropriate levels result in lung injury. Several tools are available for the evaluation of respiratory support. Chest X-ray is still prominently used to this day, although lung ultrasound is becoming incrementally prevalent in world NICUs [5]. However, both of these methods have disadvantages: ionizing radiation (X-ray), absence of real-time regional distribution as well as whole lung imaging, and limited temporal resolution. Electrical impedance tomography (EIT) stands as a relatively recent advancement in the realm of pulmonary function monitoring and ventilation guidance. This innovative tool has become increasingly prominent in clinical practice, providing practitioners with a valuable means to visualize real-time ventilation dynamics and regional distribution at the bedside. Unlike traditional imaging techniques, EIT offers continuous, dynamic insights into various aspects of respiratory function, such as compliance, ventilation map, alveolar recruitment, and regional distribution. EIT’s real-time visualization capabilities empower clinical practitioners with high temporal resolution, enabling them to promptly monitor changes and make informed decisions. This attribute is particularly crucial in intensive care settings, where swift adjustments in ventilation strategies are essential for optimal patient care [6]. 

Our study primarily aimed to assess the differences between commonly used respiratory support modes—volume-targeted conventional mechanical ventilation and non-invasive ventilation (continuous positive airway pressure (CPAP), high-flow nasal cannula (HFNC)). We evaluated end-expiratory lung impedance (EELZ) and DeltaZ, which correspond to end-expiratory lung volume (EELV) and TV, respectively, as well as global inhomogeneity index (GI index) and regional ventilation distribution. 

Our hypothesis posits that infants undergoing mechanical ventilation receive larger tidal volumes, exhibit lower EELV, and demonstrate higher levels of heterogeneity in lung function compared to those managed non-invasively. Specifically, mechanical ventilation may subject infants to larger volumes of air per breath, potentially compromising lung mechanics and increasing heterogeneity. The study proposes that these combined elements may contribute to a heightened incidence of lung injury in mechanically ventilated infants.

## 2. Materials and Methods

Trial design. This study was designed as a prospective observational trial involving data collection from a cohort of 72 infants treated in a tertiary-level Neonatal Intensive Care Unit (NICU). Approval for the study was granted by the regional ethics committee, and it was registered in advance at clinicaltrials.gov (reg. No. NCT04542096). Prior to enrollment, informed consent was obtained from the parents. The inclusion criteria involved very low birth weight (VLBW) infants (<1500 g) with gestational ages (GAs) less than 32 weeks who required respiratory support. All enrolled infants were beyond the transitional period (>72 h of age). The exclusion criteria were skin damage/abrasions on the chest at the EIT belt attachment area and significant thoracic deformity, which might influence EIT readings.

**Figure 1:**
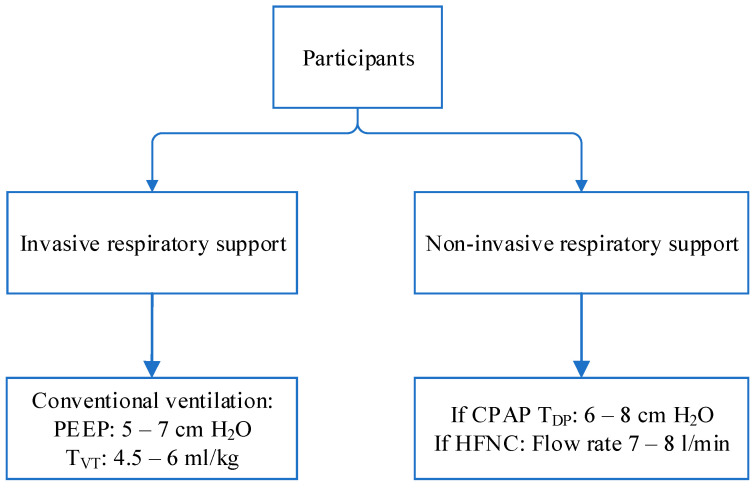
Study protocol summary. T_VT_—targeted tidal volume, T_DP_—targeted distending pressure.

Participants. Study participants were classified into 2 groups: infants receiving invasive respiratory support via endotracheal tube and infants receiving non-invasive respiratory support (CPAP or HFNC). Respiratory support systems consisted of the neonatal ventilator (Fabian HFOi, ACUTRONIC Medical Systems AG, Zürich, Switzerland), CPAP (MedinCNO (Medin Medical Innovations), and HFNC (Optiflow Junior II (Fisher & Paykel Healthcare, Auckland, New Zealand)). The fraction of inspired oxygen was clinically adjusted to provide saturation between 89 and 95% with the lowest possible FiO_2_. The study utilized the standard approach used in the center for respiratory support: mechanical ventilation was performed with conventional ventilation with an initial PEEP of 5, accompanied by tidal ventilation (volume-targeting) ranging from 4.5 to 6 mL/kg. If clinically deemed necessary, PEEP was raised to a maximum level of 7. Patients under high-frequency oscillatory ventilation were not enrolled in the study. For non-invasive respiratory support, patients on CPAP were targeted to achieve a distending pressure between 6 and 8 cm H_2_O. Those utilizing HFNC received a flow rate of 7 to 8 L per minute, irrespective of birthweight (Figure 1). All infants were in the supine position. No sedation was used on any of the participating subjects. 

Data collection: Perinatal and demographic data were obtained after enrollment. A complete antenatal corticosteroid therapy course was defined as a pregnant woman receiving two doses of betamethasone, administered 24 h apart. If the woman received only one dose, the course was considered incomplete, and if the mother did not receive any therapy, it was classified as none. Small for gestational age was characterized by a birth weight below the 10th percentile based on national growth charts. The persistence of ductus arteriosus (PDA) was evaluated by an echocardiography car performed by a pediatric cardiologist. If echocardiography displayed ductus patency, results were considered valid within a 24-h window of measurement. The hemodynamical significance of PDA (hsPDA) was defined as an echocardiographic demonstration of a ductal left-to-right shunt, volume overload as a left atrial-to-aortic root (LA/Ao) ratio of ≥1.4, and ductus size of >2 mm. Intraventricular hemorrhage (IVH) was evaluated and graded by cranial ultrasound by a pediatric neurologist. For EIT data collection, neonatal belts with 16 equally spaced electrodes were applied circumferentially around the thorax at the nipple level. The belt was placed before the desired positioning was attained. EIT data was recorded using the electrical impedance tomography device (Enlight 1800, Timpel, São Paulo, Brazil) at 50 frames per second sampling rate. Data recording commenced once the infants were comfortably positioned. Heart rate and SpO2 were manually recorded every minute from a bedside monitor (Carescape Monitor B850, GE Healthcare, Chicago, IL, USA). Before the target positioning, minimal-handling nursing care guidelines were followed [7].

Data analysis: Data analysis was performed off-line with custom design Timpel Medical’s Analysis Software (https://www.timpelmedical.com/ (accessed on 14 March 2024)) (Offline analysis tool). A period of 3 consecutive minutes was used for the analysis of the different periods. From this, 20 stable consequent, artifact-free breaths were analyzed. The following measurement periods were used: 0 min, 30 min, and 60 min. For each breath, the following were analyzed or calculated: distribution of ventilation, EELZ, DeltaZ, and heterogeneity. Regional distribution was presented as anterior and left regional ventilation fractions expressed as percentages. EELZ and DeltaZ were calculated and adjusted for body weight and expressed as arbitrary units per kilogram of body weight (AU/kg). Heterogeneity was expressed as the GI index. 

Statistical analysis: Depending on their distribution, data are expressed as mean ± SD or as median with interquartile ranges. The chi-squared test was used to compare quantitative parameters between subject groups. Normality was assessed by using the Shapiro-Wilk test. Comparative analyses were performed using the independent samples *t*-test for normally distributed data and the Mann-Whitney U test for skewed data. A linear mixed-effect model for repeated measures was used for EELZ, DeltaZ, heterogeneity, and regional ventilation and assessment to account for multiple breaths from each infant. To mitigate the issue of multiple comparisons in the analysis of the mixed-effects model, we utilized the Bonferroni correction. A *p*-value of less than 0.05 was considered to be statistically significant. Statistical analysis was performed using Minitab (LLC, version 21.0) and R statistical software (R Foundation for Statistical Computing, Vienna, Austria; version 4.1.3).

## 3. Results

**Figure 2:**
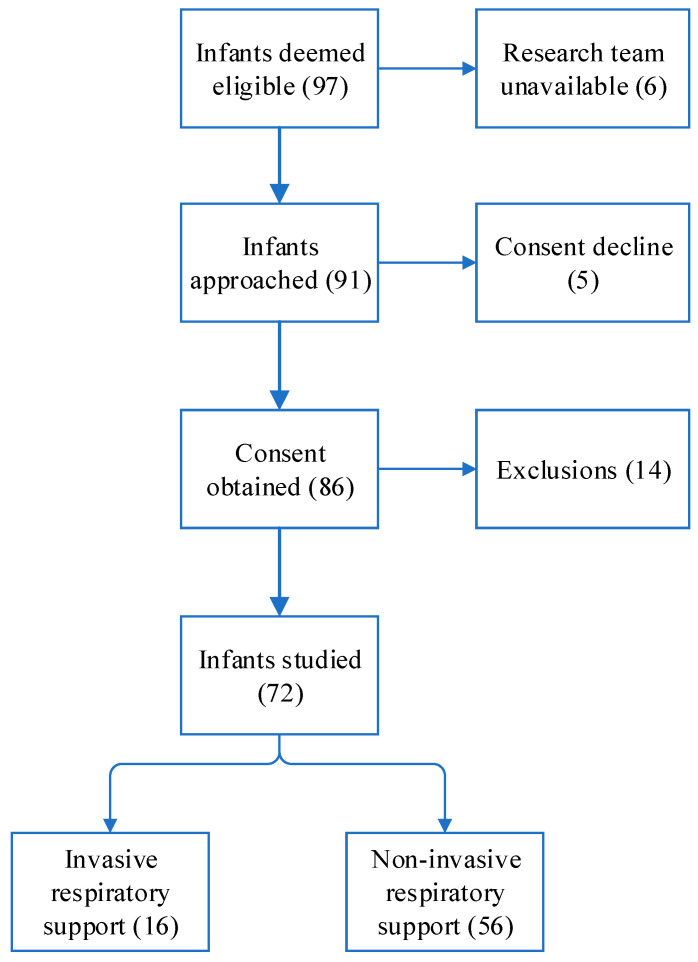
Consort diagram of the study.

The study enrolled a total of 97 infants. The research team was unavailable for 6 patients, and 19 participants, including 14 cases attributed to skin damage or abrasions and 5 instances related to consent decline or withdrawal, were subsequently excluded from the study (Figure 2). Consequently, the final analysis comprised 72 patients. All included infants were inborn infants. 

There was no substantial difference in clinical and demographic characteristics between invasive and non-invasive respiratory support receiving groups (Table 1). 

**Figure 3:**
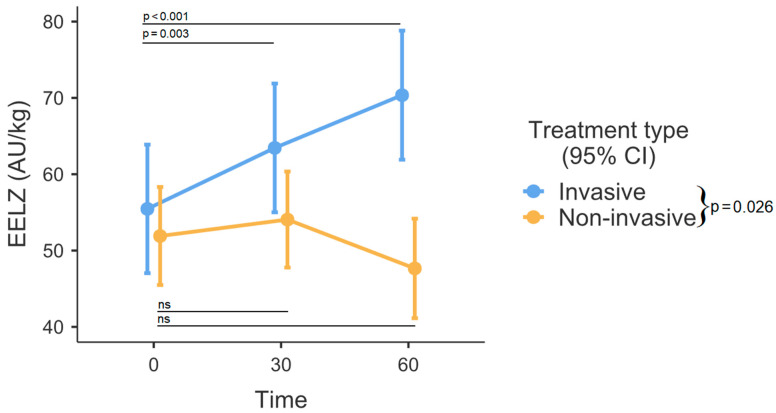
End-expiratory lung impedance (EELZ) over time between invasive and non-invasive respiratory support. Error bars represent the mean with 95% confidence intervals. Group differences are outlined in the legend. AU—arbitrary units, CI – Confidence intervals, ns – not significant.

**Figure 4:**
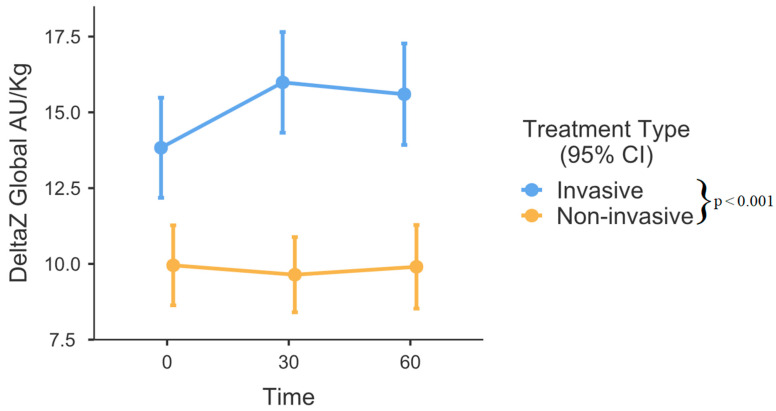
DeltaZ over time between invasive and non-invasive respiratory support. Error bars represent the mean with 95% confidence intervals. Group differences are outlined in the legend. AU—arbitrary units, CI – Confidence intervals.

**Figure 5:**
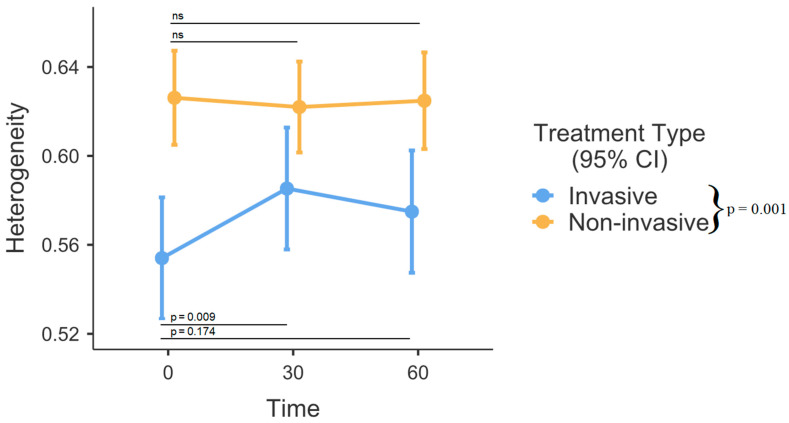
GI index over time between invasive and non-invasive respiratory support. Error bars represent the mean with 95% confidence intervals. Group differences are outlined in the legend. AU—arbitrary units, CI – Confidence intervals, ns – not significant.

No laboratory values were statistically different between the groups. Slightly over one-fourth (27.78%) of the included patients had hsPDA, while almost one-tenth (9.72%) exhibited a severe grade of IVH. The respiratory rate did not differ among the groups. However, non-invasively managed patients had a wider interquartile range (IQR). Ventilated infants had notably larger EELZ in comparison to their counterparts receiving non-invasive respiratory support (*p* = 0.026, Figure 3). Significant differences were observed among time intervals (*p* = 0.001). The analysis of fixed effects indicated notable variations in the invasive ventilation group, where end-expiratory lung impedance (EELZ) exhibited a steady increase among ventilated patients (0–30 min, *p* = 0.003, and 0–60 min, *p* < 0.001). In contrast, within the non-invasively managed group, the effects appeared somewhat random and did not reach statistical significance (*p* > 0.05). DeltaZ was also larger in the invasive respiratory support group (*p* < 0.001, Figure 4). There were no differences between various time points (0, 30, and 60 min) after position change in regard to DeltaZ. GI index was higher in the non-invasive group (*p* = 0.001, Figure 5). While the comprehensive analysis did not show statistically significant changes over time when both groups were combined (*p* = 0.109), a subgroup analysis within the invasively managed group revealed noteworthy temporal variations in the initial 30 min after monitoring commenced (0–30 min *p* = 0.009, Figure 5). However, the observed effect appeared to diminish in the later period (0–60 min, *p* = 0.174, Figure 5). The non-invasive group exhibited significantly greater dependent lung ventilation compared to the intubated patients (*p* = 0.005, Table 2). At the 30-min timepoint, both groups showed a tendency towards decreased ventilation in the anterior lung sections, although this difference did not reach statistical significance (*p* = 0.053). The left lung received less regional distribution than the right in both groups; however, in intubated patients, this was significantly more pronounced (*p* < 0.001, Table 2).

## 4. Discussion

Premature, very low birth weight infants are frequently affected by respiratory distress syndrome (RDS), which necessitates some type of respiratory support. Avoiding intubation is one of the practice strategies aimed at bronchopulmonary dysplasia (BPD) prevention. Despite technical and knowledge advancements, even “gentle” invasive ventilation remains associated with significant risks of barotrauma, volutrauma, and atelectasis [8,9]. Therefore, early implementation of non-invasive respiratory support is recommended to mitigate the risk of lung injury and BPD [10]. Consequently, recent guidelines also recommend non-invasive respiratory support as the primary and preferred mode [1]. 

Comparing invasive and non-invasive respiratory support methods poses methodological challenges. Estimating tidal volumes in infants is particularly intricate, although it can be accomplished using volumetric capnography, respiratory inductance plethysmography, or the more recent method of EIT [11,12]. Even though all the aforementioned methods (including EIT) provide only estimated information on lung capacities and volumes, this is still particularly useful in practically driven settings. We chose this method due to its portability, lack of radiation exposure, and dynamic imaging capabilities. 

Both studied groups exhibited comparable gestational and chronological ages, as well as similar birth weights. Blood pH or pCO_2_ values were almost identical between the groups. Despite the presence of severe (III–IV grade) IVH in nearly 10 percent of the cohort, none of the patients in our study were found to be anemic. The absence of anemia might be attributed to modified practices in cord clamping (delayed cord clamping). Anemia (if present) can also cause intermittent hypoxia, which in turn stimulates the respiratory center. This is an important aspect because respiratory drive is significantly influenced by these factors [13]. The aforementioned values could account for the absence of differences in respiratory rates between the groups. However, it should be noted that non-invasively managed patients had a wider interquartile range (IQR).

In our study, intubated patients were ventilated with significantly larger EELZ (which strongly correlates with end-expiratory lung volume) than those on non-invasive respiratory support [14]. This finding aligns with physiological principles, as the regulation of FRC under positive PEEP can be more effectively controlled through mechanical ventilation compared to non-invasive modes [15,16]. Other studies also demonstrated that respiratory support results in increased EELV over time [17]. In our cohort, it appeared that the observed effect was only specific to ventilated patients, while infants receiving CPAP or HFNC therapy did not exhibit the same association. On one hand, it reinforces the well-established notion that the precision of control in invasive mechanical ventilation surpasses that of non-invasive respiratory modes. However, this heightened control may inadvertently lead to the emergence of unintended intrinsic positive end-expiratory pressure (also known as occult PEEP or auto-PEEP) [18]. In neonatal practice, the conventional approach to measuring auto-PEEP presents difficulties, necessitating sedation with pharmacologic paralysis and/or esophageal manometry [19]. One emerging option is EIT-controlled PEEP titration, which is already used in adults; however, this would also require sedation [20]. Insufficient studies exist on optimal PEEP levels for RDS management, and the speculation regarding individualized PEEP titration to enhance EELV control and, consequently, mitigate VILI is currently a matter of debate [21]. 

DeltaZ (which strongly correlates with tidal volume (TV)) was also larger in ventilated patients [14]. As there were no significant differences regarding respiratory rates, it demonstrated that intubated patients received larger tidal volume per breath than those on CPAP or HFNC. Our findings are contrary to previous studies, which showed that CPAP produces similar or larger TVs than those recommended for invasive ventilation (4–6 mL/kg) by current standards of practice [22,23]. Some studies also indicate that HFNC provides TV similar to CPAP [24]. Our findings of larger EELZ and DeltaZ in intubated patients could explain why mechanical ventilation is associated with higher rates of lung injury and pulmonary air leaks [15,25,26], although some data for pneumothorax rate between therapies is conflicting [27]. Nonetheless, measuring volume and distending pressures in neonates is difficult and is known to have wide interpatient as well as intrapatient variation, which also might influence observed differences [28]. 

We also analyzed and assessed lung heterogeneity using the GI index. GI index is a quantitative measure used to assess the heterogeneity of ventilation within the lungs. This index provides information about the spatial distribution of ventilation and how evenly or unevenly air is distributed throughout the lung regions [14]. In our study, the GI index was higher in patients treated with non-invasive ventilation. The authors speculate that this result could be attributed to enhanced control of FRC and PEEP under mechanical ventilation, given the absence of a direct comparison between respiratory modes in the neonatal population. It is known from previous research that healthy infants have lower GI indexes compared to those requiring respiratory support [29]. In addition, several studies have demonstrated a reduction in heterogeneity in prone, but not supine, positioning in lower gestational age infants and children with respiratory failure [30,31]. Our findings did not show that the GI index decreased over time. This is in contrast to previous pediatric and adult studies, which showed a reduction of inhomogeneity over time [17,32]. However, it should be acknowledged that our time intervals between data collection points were relatively short in comparison to those utilized in the aforementioned studies. One should also bear in mind that, unlike ARDS, neonatal RDS is a highly homogenous disease, and thus, the comparison should be interpreted cautiously, even if the treatment effect might be similar across populations [33].

Non-dependent (anterior) lung distributions remained the same across all time periods and differed only between treatment groups. Ventilated patients had significantly larger portions of non-dependent lung ventilation. Our findings agree with previous studies, which similarly observed that spontaneous breathing favors dependent (posterior) lung [34,35,36]. One might argue that spontaneously breathing infants have more “healthy lung” tissue, which might increase strain on the non-dependent lung and, thus, reduce compliance [36]. Other studies also pointed out that mechanical ventilation negates gravity-dependent effects and favors the middle third portion of the lungs [37,38]. 

There was also a marked trend of ventilated infants to favor right-sided ventilation. Other studies demonstrated that the supine position slightly favors right region ventilation [30]. However, in our protocol, we did not specify the head position, although it is usually left in midline while supine for yet unproven intraventricular hemorrhage reduction [39]. The effect of head position has been shown to influence right-left fractional ventilation [40]. Since this was an observational trial, infants were also fed after the change of position. The feed volume in spontaneously breathing infants was likely bigger. However, the left lung ventilation did not decrease significantly in the following periods. This reinforces the understanding that feeding volume does not have a notable impact on lung compliance or significantly disrupt breathing patterns. Instances of desaturation or temporary increases in oxygen demand during or after feeding, common observations in the NICU, are likely attributed to a combination of factors, including the presence of a nasogastric tube, breathing pauses, and/or gastroesophageal reflux [41].

It is important to highlight the absence of sedation and paralysis in this study. Several studies have emphasized the significant impact of anesthesia on EIT findings. EELV, DeltaZ, lung heterogeneity, and regional ventilation distributions are all affected by anesthetic induction. The influence persists even after the post-operative period [42]. Our study, being an observational study, also provided insight into commonly employed respiratory support strategies and compared them side by side. This is, in essence, a different approach to many studies, which try to utilize EIT capabilities by providing a tailored (individualized) approach to mechanical ventilation in order to avoid complications associated with inappropriate ventilation or improve outcomes by titrating PEEP or tidal volumes where it is difficultly achieved otherwise. 

Our study has a few relevant findings: firstly, it appears that EELZ (≈EELV) and DeltaZ (≈TV) are larger even when employing gentle ventilation strategies targeting volume in comparison to those receiving non-invasive respiratory support. Secondly—lung heterogeneity was lower during invasive ventilation, although the GI index slightly increased over time. Thirdly, non-invasive respiratory support exhibits a more gravity-dependent effect on regional ventilation than mechanical ventilation. 

We acknowledge the limitations of our study. Firstly, we only examined effects on EELZ, DeltaZ, and regional distributions within a short 60-min time frame. Additionally, infants on non-invasive respiratory support were slightly older than those receiving mechanical ventilation, which could’ve influenced results. Lastly, it was an observational trial, which inherently cannot account for confounding variables influencing results. It is also noteworthy to recognize the limitations of EIT, particularly in premature infants who are prone to xerosis and breakdown of the skin, traits particularly relevant to the tiniest members of the population. Additionally, the skin is also more susceptible to thermal injury that may result from EIT belts, although this risk is typically mitigated in commercially available EIT kits by triggering alarms for increased sensor temperature. Despite this, some drawbacks of EIT are not encountered at all in the neonatal population. One contraindication of EIT is severe obesity, as it can significantly impair signal transmission through the substantial subcutaneous adipose tissue, leading to unreliable readings. Compared to adults and older children, this scenario is unfeasible in infants.

## 5. Conclusions

Our study aimed to examine the inherent disparities between infants managed invasively and their non-invasive counterparts. Our findings demonstrate that volume-targeted mechanical ventilation results in higher EELZ and DeltaZ than spontaneously breathing infants on non-invasive respiratory support. Despite this, lung heterogeneity is lower under mechanical ventilation. Our study also reaffirmed that spontaneous breathing favors dependent lungs more than mechanical ventilation.

## Figures and Tables

**Table 1:** Patient demographic, clinical, and laboratory (CBG, CBC) characteristics.

	Cohort (72)	Invasive (16)	Non-Invasive (56)	*p*-Value
Postnatal age (days)	6.0 (4.75)	6 (6.5)	6 (4)	NS
Gestational age (weeks)	28.3 (±2.2)	27.27 (±2.6)	28.68 (±2.0)	NS
Birthweight (g)	1089.9 (±268)	1016.7 (±283)	1117.2 (±261)	NS
Weight at the time of study (g)	1101.3 (±234)	1066.4 (±223)	1114.2 (±240)	NS
Male (n/%)	35 (48.61%)	8 (50%)	27 (48.21%)	NS
Inborn (n/%)	72 (100%)	16 (100%)	56 (100%)	NS
Apgar score (1 min)	8 (2.0)	8 (3.0)	8 (1.75)	NS
Apgar score (5 min)	8 (1.0)	8 (2.0)	8 (1.0)	NS
Antenatal corticosteroids				NS
Complete (n/%)	50 (69.44%)	13 (81.25%)	37 (66.07%)	
Incomplete (n/%)	6 (8.33%)	1 (6.25%)	5 (8.93%)	
None (n/%)	16 (22.22%)	2 (12.5%)	14 (25.0%)	
Chorioamnionitis (n/%)	20 (27.77%)	2 (12.5%)	18 (32.14%)	NS
Resuscitation (n/%)	19 (26.39%)	4 (25.0%)	15 (27.79%)	NS
Small for gestational age (n/%)	13 (18.06%)	1 (6.25%)	12 (21.43%)	NS
Ductus arteriosus				
PDA (n/%)	31 (43.05%)	8 (50.0%)	23 (41.07%)	NS
hsPDA (n/%)	20 (27.78%)	3 (18.75%)	17 (30.36%)	NS
IVH				
Moderate (grade 1–2) (n/%)	17 (23.61%)	2 (12.5%)	15 (26.78%)	NS
Severe (grade 3–4) (n/%)	7 (9.72%)	2 (12.5%)	5 (8.93%)	NS
Surfactant (n/%)	60 (83.33%)	15 (93.75%)	45 (80.36%)	NS
FiO_2_	0.21 (0.04)	0.24 (0.07)	0.21 (0.04)	NS
Respiratory rate	48 (20)	48 (14)	50 (25.5)	NS
Hgb	159.4 (±32.6)	151 (±36.5)	162.9 (±30.7)	NS
PLT	290 (166)	290 (130)	293.5 (189.8)	NS
Hct	45.2 (±8.8)	42.7 (±10.0)	46.2 (±8.2)	NS
pH	7.354 (±0.05)	7.351 (±0.05)	7.355 (±0.05)	NS
pCO_2_	44.1 (±7.4)	43.9 (±6.8)	42.1 (±6.8)	NS
HCO_3_	23.4 (3.4)	24.0 (5.5)	23.4 (3.3)	NS
Lac	2.0 (0.88)	2.1 (±0.8)	1.9 (0.6)	NS

The data are expressed as mean (±SD) or median (IQR). *p*-values are calculated for invasive and non-invasive respiratory support groups. CBG—capillary blood gas, CBC—complete blood count, PDA—persistent ductus arteriosus, hsPDA—hemodynamically significant ductus arteriosus, IVH—Intraventricular Hemorrhage, FiO_2_—a fraction of inspired oxygen, Hgb—hemoglobin, PLT—platelets, Hct—hematocrit, Lac—lactate, NS—not significant (*p* > 0.05).

**Table 2:** Comparison of anterior and left ventilation distribution between invasive and non-invasive respiratory support over time.

Time (Min)	0	30	60	*p*-Value
Anterior distribution (% with lower and upper 95% CI in brackets)				
Invasive	47.1 (43.9, 50.3)	44.6 (41.4, 47.9)	47.1 (43.6, 50.1)	*p* = 0.005
Non-invasive	41.4 (39.0, 44.0)	40.2 (37.8, 42.6)	41.5 (38.7, 43.9)	
Left distribution (% with lower and upper 95% CI in brackets)				
Invasive	45.3 (42.6, 47.9)	41.2 (38.4, 43.7)	41.7 (39.1, 44.5)	*p* < 0.001
Non-invasive	47.3 (45.5, 49.7)	48.8 (46.7, 50.7)	47.1 (45.6, 49.9)	

CI—Confidence interval.

## Data Availability

The datasets used and/or analyzed during the current study are available from the corresponding author upon reasonable request.
